# Development of a heat‐stable alkaline phosphatase reporter system for *cis*‐regulatory analysis and its application to 3D digital imaging of *Xenopus* embryonic tissues

**DOI:** 10.1111/dgd.12919

**Published:** 2024-03-04

**Authors:** Kiyo Sakagami, Takeshi Igawa, Kaori Saikawa, Yusuke Sakaguchi, Nusrat Hossain, Chiho Kato, Kazuhito Kinemori, Nanoka Suzuki, Makoto Suzuki, Akane Kawaguchi, Haruki Ochi, Yuki Tajika, Hajime Ogino

**Affiliations:** ^1^ Department of Animal Bioscience Nagahama Institute of Bio‐Science and Technology Nagahama Japan; ^2^ Amphibian Research Center, Graduate School of Integrated Sciences for Life Hiroshima University Hiroshima Japan; ^3^ Department of Pharmaceutical Sciences North South University Dhaka Bangladesh; ^4^ Department of Genomics and Evolutionary Biology National Institute of Genetics Shizuoka Japan; ^5^ Institute for Promotion of Medical Science Research, Faculty of Medicine Yamagata University Yamagata Japan; ^6^ Department of Radiological Technology Gunma Prefectural College of Health Sciences Maebashi Japan

**Keywords:** imaging, placental alkaline phosphatase (PLAP), reporter, transgenesis, *Xenopus*

## Abstract

*Xenopus* is one of the essential model systems for studying vertebrate development. However, one drawback of this system is that, because of the opacity of *Xenopus* embryos, 3D imaging analysis is limited to surface structures, explant cultures, and post‐embryonic tadpoles. To develop a technique for 3D tissue/organ imaging in whole *Xenopus* embryos, we identified optimal conditions for using placental alkaline phosphatase (PLAP) as a transgenic reporter and applied it to the correlative light microscopy and block‐face imaging (CoMBI) method for visualization of PLAP‐expressing tissues/organs. In embryos whose endogenous alkaline phosphatase activities were heat‐inactivated, PLAP staining visualized various tissue‐specific enhancer/promoter activities in a manner consistent with green fluorescent protein (GFP) fluorescence. Furthermore, PLAP staining appeared to be more sensitive than GFP fluorescence as a reporter, and the resulting expression patterns were not mosaic, in striking contrast to the mosaic staining pattern of β‐galactosidase expressed from the *lacZ* gene that was introduced by the same transgenesis method. Owing to efficient penetration of alkaline phosphatase substrates, PLAP activity was detected in deep tissues, such as the developing brain, spinal cord, heart, and somites, by whole‐mount staining. The stained embryos were analyzed by the CoMBI method, resulting in the digital reconstruction of 3D images of the PLAP‐expressing tissues. These results demonstrate the efficacy of the PLAP reporter system for detecting enhancer/promoter activities driving deep tissue expression and its combination with the CoMBI method as a powerful approach for 3D digital imaging analysis of specific tissue/organ structures in *Xenopus* embryos.

## Graphical abstract

In this study, we (1) optimized the conditions for chromogenic detection of placental alkaline phosphatase (PLAP) (alkaline phosphatase, placental type) expression for use as a transgenic reporter in *Xenopus* embryos, (2) investigated the sensitivity and responsiveness of PLAP to the *cis*‐regulatory elements of various genes, and (3) analyzed embryos expressing PLAP in specific tissues using the correlative light microscopy and block‐face imaging (CoMBI) method. The results of these experiments demonstrate the efficacy of the PLAP reporter system for detecting enhancer/promoter activities driving deep tissue expression and its combination with the CoMBI method as a powerful approach for 3D digital imaging analysis of specific tissue/organ structures in opaque *Xenopus* embryos.

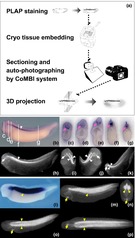

## 1 INTRODUCTION

Analysis of tissue/organ structures expressing a gene of interest in developing embryos is essential for understanding how morphogenesis proceeds and how genes control morphogenesis. Classically, such analyses have been performed by making serial sections of histochemically or immunologically stained specimens followed by imaginative reconstruction of 3D tissue/organ structures in the mind of the researcher. However, the situation has been dramatically changed by introducing fluorescent proteins for in vivo tissue/organ labeling and developing fluorescence microscopy techniques for 3D imaging of deep tissues (Whitehead et al., 2017). Confocal laser scanning microscopy (CLSM) is popular for high‐spatial‐resolution analysis of relatively thin specimens, light‐sheet fluorescence microscopy (LSFM) is used for high‐speed analysis of thicker specimens with low photo‐toxicity, and two‐photon excitation microscopy is used for the analysis of thick specimens to which CLSM and LSFM are not applicable. In vertebrate model systems, zebrafish is suitable for such in vivo fluorescence microscopy analyses, owing to the inherent transparency of its embryos and ease in generating transgenic reporter lines expressing fluorescent proteins in specific tissues (Abu‐Siniyeh & Al‐Zyoud, 2020). Especially, the use of genome editing technology in transgenic zebrafish followed by in vivo fluorescence microscopy has seen remarkable success in high‐throughput analysis of genotype–phenotype correlations in the developing heart and central nervous system (Naert & Vleminckx, 2018). However, since the knowledge available from a single model system is limited, it is essential to develop efficient 3D imaging techniques in other vertebrate model systems where high‐throughput transgenesis and genome editing technologies are available, in order to reveal conserved and species‐specific aspects of vertebrate development.

As with zebrafish, the clawed frogs *Xenopus laevis* and *Xenopus tropicalis* are popular model systems for studying vertebrate development (Sive et al., 2000). *X. laevis* has been traditionally used for embryological experiments by virtue of its large embryos, which facilitate surgical manipulation and microinjection experiments, though its allotetraploid genome and long generation time (around 1 year under standard husbandry conditions) has hindered the use of this species for genetic experiments. *X. tropicalis* has been launched as an alternative frog system suitable for genetics, because of its compact diploid genome (1.7 Gb, nearly half that of the mouse genome) and shorter generation time (less than 6 months) (Amaya et al., 1998; Harland & Grainger, 2011; Ogino & Ochi, 2009). The *X. tropicalis* genome shares substantial synteny with the human genome over major parts of large chromosomes and possesses orthologs of 79% of identified human disease genes, highlighting the advantages of this species for disease modeling studies (Hellsten et al., 2010). Regarding transgenesis in *Xenopus* (reviewed in Ogino & Ochi, 2009), one can quickly generate hundreds of founder animals with non‐mosaic transgene expression by the sperm nuclear transplantation method or the I‐*Sce*I meganuclease method (Kroll & Amaya, 1996; Ogino et al., 2006a, 2006b; Pan et al., 2006). Genome editing technology also works with high efficiency; CRISPR/Cas9‐mediated gene knockout has become one of the standard experimental techniques in the *Xenopus* system (Nakayama et al., 2014, 2020; Ochi et al., 2017; Suzuki et al., 2018; Tandon et al., 2017; Tanouchi et al., 2022).

Despite these advantages as a model system, the application of 3D imaging techniques for tissue/organ analysis has been limited in *Xenopus*. Although in vivo fluorescence microscopy in this species has contributed a lot to understanding mechanisms of morphological cell movements at the gastrula/neurula stages and organ development at later stages, most of these studies were performed with a focus on surface structures, explant cultures, or post‐embryonic transparent tadpoles (Chu et al., 2020; Kolker et al., 2000; Periasamy et al., 1999; Robb & Wylie, 1999; Suzuki et al., 2019). This limitation is due to the fact that *Xenopus* embryonic cells contain a lot of refractile yolk platelets, which hamper the fluorescence imaging of deep structures. It is possible to visualize deep tissues/organs by performing chromogenic whole‐mount in situ hybridization or immunostaining followed by clearing light‐scattering cellular components with a benzyl benzoate/benzyl alcohol mixture (Sive et al., 2000). However, it is not easy for probes and antibodies to penetrate deep into tissues, especially in late‐stage embryos and tadpoles, and techniques for reconstructing 3D digital images from such whole‐mount samples have not yet been systematically established.

In this study, we attempted to overcome the limitation in the 3D imaging analysis of *Xenopus* embryos by adapting the correlative light microscopy and block‐face imaging (CoMBI) method (Tajika et al., 2017). This method collects serial block‐face images of a frozen specimen using a consumer‐grade, digital single‐lens reflex camera and a standard cryostat and reconstructs a 3D image of the specimen by stacking the collected images in a computer. The method effectively works with naturally colored or histochemically stained, relatively large specimens, such as whole mouse embryos and juvenile zebrafish stained with hematoxylin or tannic acid, and does not require expensive, specialized fluorescence microscopes (Ishii et al., 2021; Makanae et al., 2020; Sutrisno et al., 2023). To visualize target tissues for 3D imaging analysis with the CoMBI method, we chose to use a heat‐stable, chromogenic reporter, human placental alkaline phosphatase (generally referred to as PLAP; the official symbol is ALPP) (Deprimo et al., 1996; Fekete & Cepko, 1993), in place of green fluorescent protein (GFP), which has been widely used in *Xenopus* transgenesis (Ogino & Ochi, 2009). A previous study has shown that PLAP expression can label neural tissues in transgenic *Xenopus* tadpoles (Huang et al., 2007). Here, we investigated heat treatment conditions for detecting PLAP expression without background staining of endogenous alkaline phosphatase (AP) activity. We then evaluated the detection sensitivity and responsiveness of the PLAP gene using various *cis*‐regulatory elements to use it as a high‐throughput chromogenic reporter. Finally, we successfully applied this PLAP reporter system to the CoMBI method (PLAP‐CoMBI) for convenient 3D digital imaging of embryonic tissues in *Xenopus*.

## 2 MATERIALS AND METHODS

### 2.1 Fixation, heat treatment, and AP staining of embryos

**FIGURE 1:**
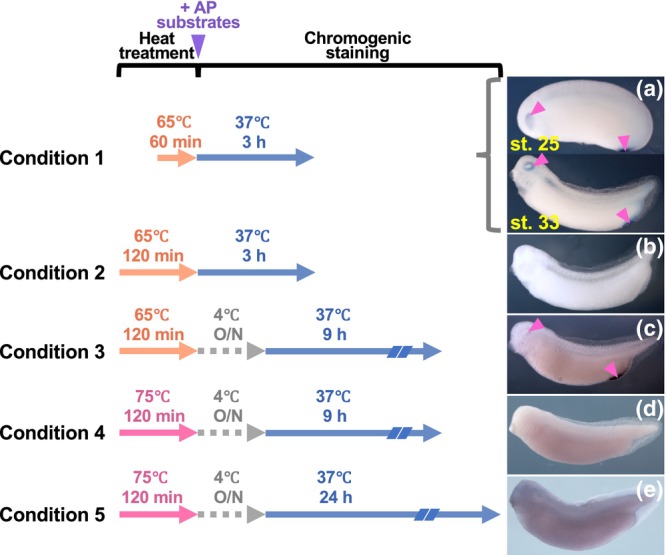
Identification of optimum heat treatment conditions to inactivate endogenous alkaline phosphatase in *Xenopus*. (a, b) Fixed wild‐type embryos (stages 25 and/or 33) were treated at 65°C for 60 or 120 min and then subjected to a chromogenic alkaline phosphatase (AP) reaction at 37°C for 3 h (Conditions 1 and 2, respectively). (c) Fixed wild‐type embryos (Stage 33) were treated at 65°C for 120 min and then subjected to a chromogenic AP reaction at 4°C overnight, followed by the AP reaction at 37°C for 9 h (Condition 3). (d, e) Fixed wild‐type embryos (Stage 33) were treated at 75°C for 120 min and then subjected to a chromogenic AP reaction at 4°C overnight, followed by the AP reaction at 37°C for 9 or 24 h (Conditions 4 and 5, respectively). Representative embryos are shown for Conditions 1 to 5. Magenta triangles indicate AP staining in the eye and tissues surrounding the anus. The purple triangle indicates the timing for providing AP substrates to the heat‐treated embryos.

*X. laevis* embryos were fixed with MEMFA (0.1 M MOPS [pH 7.4], 2.0 mM EGTA, 1.0 mM MgSO_4_, 3.7% formaldehyde) for 1 h at room temperature and then washed three times (10 min each wash) in PTw (1× PBS, 0.1% Tween‐20) using a nutator. The embryos were subsequently incubated under a series of conditions indicated in Figure 1 to examine the effects on endogenous AP activity. After this treatment, the embryos were washed twice (5 min per wash) in AP buffer (100 mM Tris–HCl [pH 9.5], 50 mM MgCl_2_, 100 mM NaCl, 0.1% Tween 20) at room temperature and then incubated in BM‐Purple (Roche 11442074001) supplemented with 0.1% Tween 20 for chromogenic detection of endogenous AP and/or exogenous PLAP activity. As shown in Figure 1, this chromogenic reaction was performed with or without the overnight pre‐incubation step at 4°C to examine whether this pre‐incubation stimulates penetration of BM‐Purple into the embryos and increases the sensitivity of the subsequent chromogenic reaction at 37°C. We found that increasing the MEMFA fixation time to more than 1 h apparently decreased PLAP activity in transgenic embryos.

After the chromogenic reaction, the embryos were processed as embryos subjected to standard whole‐mount in situ hybridization analysis (Sive et al., 2000). Briefly, the embryos were treated with Bouin's fixative overnight, washed five or six times with a 70% ethanol–water mixture (more than 1 h per wash), and then rehydrated by sequential washes with a 50% ethanol–water mixture, a 25% ethanol–75% PTw mixture, and PTw. The rehydrated embryos were placed in bleaching solution (1% hydrogen peroxide, 5% formamide, 0.5× SSC) and gently rocked on aluminum foil under fluorescent light until the melanin pigment disappeared. The bleached embryos were stored in 70% ethanol and subjected to subsequent analyses.

### 2.2 Comparative genome analysis

We have previously identified CNS3 and CNS5 as the non‐coding sequences conserved in the 5′ flanking regions of human, mouse, and *X. tropicalis six3* genes, and we have shown that they recapitulate the eye‐ and forebrain‐specific expression in late neurula embryos when linked to a heterologous basal promoter (Hellsten et al., 2010). Comparative genome analysis was performed by downloading the 41‐kb genomic sequence of the *X. tropicalis six3* locus containing CNS3 and CNS5 (v10.0, chr5: 19,105,574–19,146,162) and its orthologous sequences in *Nanorana parkeri* (nanPar1, KN905911v1: 2,222,473–2,260,805), *Anolis carolinensis* (anoCar2, GL343284: 1,362,377–1,430,990), *Gallus gallus* (galGal5, chr3: 25,878,705–25,902,419), *Mus musculus* (mm39, chr17: 85,909,115–85,932,674), *Homo sapiens* (hg38, chr2: 44,922,482–44,946,160), and *Takifugu rubripes* (fr3, chr4: 4,737,903–4,754,537) from Xenbase (http://www.xenbase.org/, RRID:SCR_003280) and the UCSC Genome Browser (http://genome.ucsc.edu.) and aligning them using MultiPipMaker and PipHelper (Schwartz, 2003).

### 2.3 Plasmid constructs

A basal GFP reporter plasmid used for transgenic experiments, ISceI‐pBSII SK+eGFP, was generated by inserting an eGFP‐poly(A) cassette derived from pEGFP‐1 (Clontech) into ISceI‐pBSII SK+ (a gift from Jochen Wittbrodt) (Ogino et al., 2006a; Thermes et al., 2002). We used this enhanced GFP (eGFP) derived from pEGFP‐1 as a GFP gene in this study. A basal PLAP reporter plasmid, ISceI‐pBSII SK+PLAP, was generated by inserting a PLAP‐coding sequence derived from RCASBP‐AP(A) (CC#37) (a gift from Connie Cepko, Addgene plasmid #15160; http://n2t.net/addgene:15160; RRID:Addgene_15160) (Fekete & Cepko, 1993) and a poly(A) cassette derived from pCS2+ (Rupp et al., 1994) into ISceI‐pBSII SK+.

For the construction of reporter plasmids carrying *six3 cis*‐regulatory elements, CNS3, CNS5, and a proximal promoter region of *six3* were amplified from *X. tropicalis* (Nigerian‐A strain; Igawa et al., 2015) genomic DNA by PCR using the following primers (linker sequences are underlined): CNS3, 5′‐GTACGGATCCATCTCCGCAATAAGCCCCTGAAC‐3′ and 5′‐GTACAGATCTCACTAAATTGGCGACTCTGCCTTC‐3′; CNS5, 5′‐GTACGGATCCTCCCATTCAGGCAGAAGCAAATGG‐3′ and 5′‐GTACAGATCTGGAACACACAAAGCGGCTACTTATC‐3′; the proximal promoter region, 5′‐GATCGAATTCAGCAAGTGGAGCAGGAATATAGCG‐3′ and 5′‐GATCAAGCTTGAGAAAGAAGCAGCGGCACTAAC‐3′. The amplified DNA fragments were cloned into ISceI‐pBSII SK+eGFP and ISceI‐pBSII SK+PLAP to generate *Tg(Xtr.six3:eGFP)* and *Tg(Xtr.six3:ALPP)* and verified by sequencing. To name the plasmid constructs newly generated in this study, we used ALPP, the official gene symbol of PLAP, following the current *Xenopus* transgene nomenclature guideline (https://www.xenbase.org/gene/static/tgNomenclature.jsp). A series of reporter plasmids carrying an *X. laevis EF‐1 alpha* (*eef1a1*) promoter, *Tg(Xla.eef1a1:ALPP)*, *Tg(Xla.eef1a1:lacZ)*, and *Tg(Xla.eef1a1:lagoZ)*, were generated from IS‐XeX‐GFP (Ogino et al., 2006b) by replacing its GFP (eGFP) sequence with the PLAP sequence derived from RCASBP(A)PLAP, a *lacZ* sequence derived from pCH110 (Amersham), or a *lagoZ* sequence derived from pEF1αLagoZ (a gift from Jean‐François Nicolas) (Chevalier‐Mariette et al., 2003), respectively, and then verified by sequencing. Reporter plasmids, *Tg(Xtr.tubb2b:eGFP)* and *Tg(Xtr.tubb2b:ALPP)*, which carry a 3.45‐kb *X. tropicalis beta‐tubulin* (*tubb2b*) promoter (v10.0, chr5: 87715122.87718567), were generated by PCR amplification of a genomic fragment from *X. tropicalis* DNA using primers 5′‐GATCGAATTCGCAGGAAATTGCCTGAGTAATTCTGATGG‐3′ and 5′‐GATCGTCGACGCGGTGTAAATCAGTGGATGTTGTAGC‐3′, followed by cloning of the resulting amplicons into ISceI‐pBSII SK+eGFP and ISceI‐pBSII SK+PLAP, respectively, and then verification by sequencing. A reporter plasmid carrying an *X. laevis cardiac alpha‐actin* (*actc1*) promoter, *Tg(Xla.actc1:ALPP)*, was generated by cloning of a 0.6‐kb proximal promoter (an *Eco*RI–*Sal*I fragment) derived from pCarGFP (a gift from Tim J. Mohun; Latinkić et al., 2002) into ISceI‐pBSII SK+PLAP.

### 2.4 Transgenic reporter assay

Transgenic *Xenopus* embryos were generated by a sperm nuclear transplantation method (Sive et al., 2000), with minor modifications in which I‐*Sce*I meganuclease and the reporter plasmid were co‐injected with sperm nuclei into unfertilized eggs to increase the transgenesis efficiency. The manipulated embryos were cultured until the early neurula, tailbud, or tadpole stage, and normally developed embryos were subjected to epifluorescence microscopy for detecting GFP expression, AP staining for PLAP expression, or X‐gal staining for *lacZ* or *lagoZ* expression. The X‐gal staining was performed as described previously (Sive et al., 2000), and the resulting embryos were bleached, as were the embryos subjected to AP staining.

### 2.5 CoMBI analysis

For 3D imaging of the transgenic embryos (*Tg(Xtr.tubb2b:ALPP)* and *Tg(Xla.actc1:ALPP)*), we embedded them in frozen blocks as right circular cylinders 14 mm in diameter or regular square (10 × 10 mm) columns and mounted each block to a Leica CM3050S cryostat (Leica Microsystems K. K., Tokyo, Japan). In front of this cryostat, we set a digital single‐lens reflex camera (Nikon D810, Nikon, Tokyo, Japan) with a macro lens (Tamron AF180 mm F/3.5 Di LD [IF] MACRO1:1; Tamron, Saitama, Japan) on a tripod (Husky #1003; Toyo Trading, Kyoto, Japan) with a geared head (Manfrotto 410; Manfrotto Distribution K. K., Tokyo, Japan). The camera was connected to the core control device of the system, which was prepared according to Tajika et al. (2017) and the instruction manual of the CoMBI method (https://combi-3d.github.io/manual/). This control device coordinates the automated continuous sectioning of the frozen block by the cryostat and the acquisition of serial images of sectioned block planes by the camera. The resulting images were processed using ImageJ v1.47 64‐bit (NIH, Bethesda, MA) following Tajika et al. (2017). The processed images were imported as Z‐stack images in Zen 2.6 (blue edition) (Carl Zeiss Microscopy GmbH, Oberkochen, Germany) for reconstruction into 3D images.

## 3 RESULTS

### 3.1 Optimization of heat treatment conditions for inactivating endogenous alkaline phosphatase activity of *Xenopus* embryos

Previously, Huang et al. (2007) fixed *X. laevis* tailbud embryos and tadpoles with formaldehyde and treated them at 65°C for 60 min to inactivate the endogenous AP before subjecting them to an AP chromogenic reaction for up to 30 min to detect expression of exogenous PLAP. However, when we treated wild‐type late‐neurula and tailbud embryos as in Huang et al. (2007) and subjected them to 3 h of AP chromogenic reaction at 37°C, the resulting embryos exhibited apparent staining signals in the eye and tissues surrounding the anus (Figure 1a, Condition 1). Moreover, the staining appeared more intense in the tailbud embryos than in the neurula embryos. These results indicate that heat treatment at 65°C for 60 min is insufficient for the complete inactivation of endogenous AP at these stages, which appears to be more resistant as development proceeds.

Since such residual AP activity could result in background noise when detecting exogenous PLAP expression, we investigated the effects of various heat treatment conditions on endogenous AP activity. When we extended the heating time from 60 to 120 min without increasing the temperature, the treated tailbud embryos showed no detectable staining after 3 h of AP reaction at 37°C (Figure 1b, Condition 2). However, when we pre‐incubated the treated embryos with AP substrates overnight at 4°C to increase penetration of the substrates into deep tissues before subjecting them to 9 h of AP reaction at 37°C, the resulting embryos exhibited staining signals again in the eye and tissues surrounding the anus (Figure 1c, Condition 3). Since these results suggest that a temperature of 65°C is not high enough for effective inactivation of endogenous AP, we next examined the efficacy of a higher temperature. When we treated tailbud embryos at 75°C for 120 min, the embryos exhibited no detectable staining after overnight pre‐incubation with the AP substrates at 4°C, followed by 9 h, or even 24 h, of AP reaction at 37°C (Figure 1d, Condition 4; Figure 1e, Condition 5). Based on these results, we concluded that heat treatment at 75°C for 120 min was sufficient for reducing AP activity from tailbud embryos and also from earlier‐stage embryos with lower AP activity to non‐detectable levels.

### 3.2 Comparison of detection sensitivities between GFP fluorescence and PLAP staining as transgenic reporters

**FIGURE 2:**
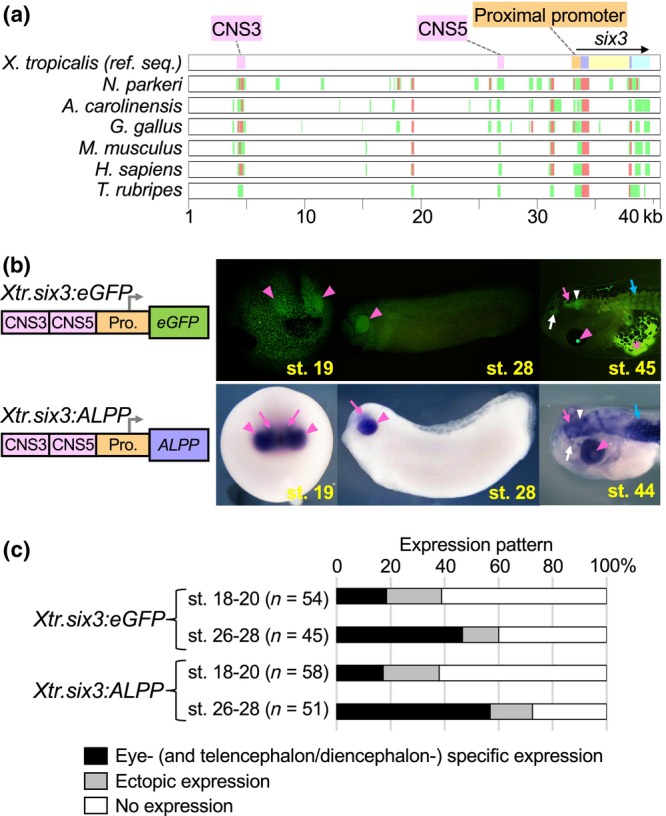
Comparison of detection sensitivities between green fluorescent protein (GFP) fluorescence and placental alkaline phosphatase (PLAP) staining using *six3 cis*‐regulatory elements. (a) Schematic representation of an alignment of a genomic sequence corresponding to the *Xenopus tropicalis six3* gene and its upstream region with the orthologous genomic sequences of *Nanorana parkeri*, *Anolis carolinensis*, *Gallus gallus*, *Mus musculus*, *Homo sapiens*, and *Takifugu rubripes*. In the reference *X. tropicalis* sequence, the coding regions, intron, and 3' untranslated region (UTR) of *six3* are shaded in purple, yellow, and cyan, respectively. Previously identified conserved enhancers (CNS3 and CNS5) and a conserved proximal promoter region including the 5'UTR of *six3* are shaded in pink and orange, respectively. Sequences with more than 75% identity to the orthologous *X. tropicalis* sequence are shown in red, and sequences with 50% to 75% identity are shown in green in the six aligned species sequences. (b) GFP and PLAP expression driven by the enhancers CNS3 and CNS5 and the proximal promoter of *six3*. Upper panels show the injected GFP reporter construct, representative embryos exhibiting GFP fluorescence in the eye primordia at the neurula and tailbud stages (pink triangles, stages 19 and 28), and a representative tadpole (stage 45) exhibiting GFP fluorescence in the eye (pink triangle), telencephalon (pink arrow), diencephalon (white triangle), nasal placodes (white arrow), and somite (blue arrow). In the eye, the pigment epithelium mostly covers the neural retina, which obscures fluorescence signals from the neural retina. A long‐pass filter was used in GFP epifluorescence microscopy to visualize the whole embryo shape with the yellowish autofluorescence of the yolk (stages 19 and 28). The pink asterisk indicates autofluorescence in the intestine (stage 45). Lower panels show the injected PLAP reporter construct, representative embryos exhibiting PLAP staining signals in the eye primordia (pink triangles) and in the telencephalon and diencephalon primordia (pink arrows) at the neurula and tailbud stages (stages 19 and 28), and a representative tadpole (stage 44) exhibiting PLAP staining signals in the eye (pink triangle), telencephalon (pink arrow), diencephalon (white triangle), nasal placodes (white arrow), and somite (blue arrow). (c) Bar graphs summarizing the transgenic reporter experiments. For each construct, reproducible expression patterns consistent with the representative examples shown in (b) were scored as “eye‐ (and telencephalon/diencephalon‐) specific expression” at the indicated stages. Expression patterns that were reproducible in <5% of the analyzed embryos were scored collectively as “ectopic expression.”

To evaluate the detection sensitivity of PLAP as a transgenic reporter with the optimized heat treatment condition, we chose to compare the expression of PLAP with that of a widely used reporter, GFP, under the control of identical *cis*‐regulatory elements in *Xenopus* embryos. For this purpose, we focused on CNS3 and CNS5, which are eye‐ and forebrain‐specific enhancers of *six3* that have been previously identified by comparing the genomic sequence of the human *six3* locus with its orthologous sequences in mouse, pufferfish, and *X. tropicalis* (Hellsten et al., 2010). In the present study, we compared the *X. tropicalis* genomic sequence of a 40‐kb segment (encompassing CNS3, CNS5, and *six3* exons) with the orthologous segments in the Tibetan frog (*N. parkeri*), lizard (*A. carolinensis*), chicken (*G. gallus*), mouse (*M. musculus*), human (*H. sapiens*), and pufferfish (*T. rubripes*) genomes using the MultiPipMaker alignment tool (Figure 2a). In addition to CNS3 and CNS5, this analysis revealed that a 0.74‐kb proximal promoter sequence (*X. tropicalis* genome assembly v10.0, chr5: 19,138,557–19,139,299) was also conserved in these species.

Hence, we cloned the *X. tropicalis* CNS3, CNS5, and 0.74‐kb proximal promoter together into either a GFP or a PLAP reporter plasmid to generate *Tg(Xtr.six3:eGFP)* or *Tg(Xtr.six3:ALPP)*, respectively. When *Tg(Xtr.six3:eGFP)* was used for transgenesis, GFP fluorescence was first detected in the eye primordia of 19% of the injected embryos at neurula stages (*n* = 10/54, stages 18–20) (Figure 2b, upper left panel; Figure 2c, top bar graph). As development proceeded, the fraction of embryos exhibiting eye primordium‐specific GFP fluorescence increased, reaching 47% at tailbud stages (*n* = 21/45, stages 26–28) (Figure 2b, upper middle panel; Figure 2c, second bar graph). At the tadpole stage (stage 45), GFP fluorescence was observed through the eye lens and in the telencephalon, diencephalon, nasal placodes, and somite (Figure 2b, upper right panel).

When embryos were injected with the *Tg(Xtr.six3:ALPP)* construct, fixed at neurula stages (stages 18–20), and subjected to the optimized heat treatment followed by AP chromogenic reaction, AP staining signals were detected in the eye primordia of 17% of the resulting embryos (*n* = 10/58) (Figure 2b, lower left panel; Figure 2c, third bar graph). In addition to the eye primordia, AP staining was detected in the telencephalic and diencephalic primordia of these embryos. When the injected embryos were fixed at tailbud stages (stages 26–28), 57% of them showed AP staining in the eye primordia as well in the telencephalic and diencephalic primordia (*n* = 29/51) (Figure 2b, lower right panel; Figure 2c, fourth bar graph). At the tadpole stage (stage 44), AP staining was detected in the eye, telencephalon, diencephalon, nasal placodes, and somite (Figure 2b, lower right panel). Since we bleached the skin and eye pigments of the tadpoles, AP staining was detected throughout their eyes, providing contrast to the tadpoles injected with *Tg(Xtr.six3:eGFP)*, whose GFP fluorescence in the eye appeared to be largely obscured by pigments. We note that PLAP expression was detected in the telencephalic and diencephalic primordia from neurula stages. In contrast, GFP fluorescence was detected in the telencephalon and diencephalon only at later stages when the embryos developed into transparent tadpoles, whereas previous in situ hybridization analysis detected endogenous *six3* mRNA expression in the eye, as well as telencephalic and diencephalic primordia from neurula stages (Zhou et al., 2000). These results suggest that AP staining is more sensitive than GFP fluorescence as a transgenic reporter. This difference in detection sensitivity may be due to the better contrast of AP staining signals compared to GFP fluorescence in the opaque embryos. GFP and PLAP expression in tadpole somites is specific to the *cis*‐regulatory elements used in this study, but does not appear to reflect endogenous *six3* expression, which is absent from somites (Zhou et al., 2000).

Chi‐square tests indicated no significant difference between the ratio of embryos with eye‐specific GFP signals and the ratio of embryos with eye‐, telencephalon‐, and diencephalon‐specific AP signals in the manipulated embryos at the neurula and tailbud stages (*p* > .05), indicating little difference in the detection timing of PLAP and GFP as a reporter. Chi‐square tests also indicated no significant difference between the ratio of embryos with non‐reproducible ectopic GFP signals (*n* = 11/54 at stages 18–20, *n* = 6/45 at stages 26–28) and the ratio of embryos with ectopic AP signals (*n* = 12/58 at stages 18–20, *n* = 8/51 at stages 26–28) in the manipulated embryos at the neurula and tailbud stages (*p* > .05), indicating that the PLAP gene does not possess any intrinsic activity to stimulate ectopic expression.

### 3.3 Comparison of PLAP expression with GFP and *lacZ* expression in combination with other promoters

**FIGURE 3:**
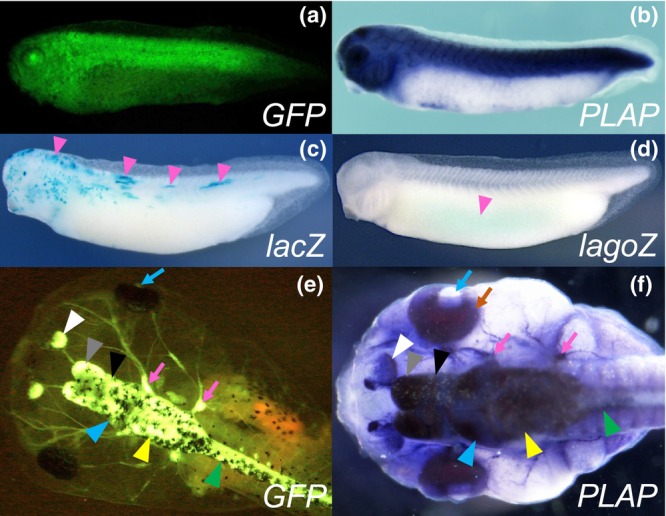
Comparison of green fluorescent protein (GFP), placental alkaline phosphatase (PLAP), *lacZ*, and *lagoZ* expression driven by an *eef1a1* or *tubb2b* promoter. (a–d) Representative expression patterns of GFP, PLAP (ALPP), *lacZ*, and *lagoZ* driven by an *eef1a1* promoter in tailbud embryos. The expression of GFP and PLAP was visible as green fluorescence and AP staining, respectively. Pink triangles indicate part of the X‐gal staining signals. (e, f) Representative expression patterns of GFP and PLAP (ALPP) driven by a *tubb2b* promoter in tadpoles. White, gray, black, blue, yellow, and green triangles indicate GFP fluorescence (e) or AP staining signals (f) in the olfactory epithelium, telencephalon, diencephalon, midbrain, hindbrain, and spinal cord, respectively. Pink arrows indicate GFP fluorescence (e) or AP staining signals (f) in the cranial nerves. (f) The purple AP staining in the eye (brown arrow) appears to merge with the brownish color of the residual retinal pigment that remained after the bleaching reaction. Given the absence of AP staining in the lens (f, blue arrow), the GFP fluorescence observed through the lens likely originated from the neural retina covered by the pigment epithelium (e, blue arrow).

To examine whether PLAP can respond to various promoters and visualize their activities, we compared PLAP expression patterns driven by an *X. laevis eef1a1* promoter with GFP or *lacZ* expression patterns driven by the same promoter in transgenic *X. laevis* embryos at tailbud stages (stages 33/34). The *lacZ* gene is popular in mammalian transgenesis as a chromogenic reporter gene, and its misexpression by RNA injection is a standard method for cellular lineage labeling in *Xenopus* embryos (Sive et al., 2000). As previously reported, the embryos injected with a GFP reporter construct carrying the *eef1a1* promoter (IS‐XeX‐GFP) showed intense GFP fluorescence in the dorsal tissues (*n* = 15/55, Figure 3a) (Ogino et al., 2006b). Consistent with this GFP expression pattern, the embryos injected with a PLAP reporter construct carrying the same *eef1a1* promoter (*Tg(Xla.eef1a1:ALPP)*) also showed intense AP staining in the dorsal tissues (*n* = 14/47, Figure 3b). In contrast, the embryos injected with a *lacZ* reporter construct carrying the same *eef1a1* promoter (*Tg(Xla.eef1a1:lacZ)*) showed only spotty X‐gal staining in the dorsal tissues (*n* = 17/47, Figure 3c). Since such aberrant *lacZ* expression might be due to the methylation of its CpG sites, we replaced *lacZ* with *lagoZ*, a CpG‐null derivative of *lacZ*, to use for transgenesis in combination with the *eef1a1* promoter (Chevalier‐Mariette et al., 2003). In embryos injected with the resulting construct (*Tg(Xla.eef1a1:lagoZ)*), no spotty X‐gal staining was detected, but only faint staining was detected in the ventral tissues (*n* = 12/40, Figure 3d). The remaining sibling embryos scored as “non‐expressors” showed no detectable X‐gal staining in their ventral tissues (*n* = 28/40), suggesting that the X‐gal staining in the 12 embryos scored as “expressors” is not a non‐specific background signal.

We also compared GFP and PLAP expression patterns driven by an *X. tropicalis tubb2b* promoter. In both transgenic tadpoles injected with either a GFP construct (*Tg(Xtr.tubb2b:eGFP)*) or a PLAP construct (*Tg(Xtr.tubb2b:ALPP)*), the nervous tissues, such as the olfactory epithelium, telencephalon, diencephalon, midbrain, hindbrain, spinal cord, and cranial nerves, were clearly visualized with GFP fluorescence or AP staining (Figure 3e,f). As with the transgenic experiments using the *six3 cis*‐regulatory elements, PLAP expression was detected throughout the eye, but GFP fluorescence in the pigmented eye was observed only through the lens. These results obtained with the *cis*‐regulatory elements of *six3*, *eef1a1*, and *tubb2b* indicate that PLAP can visualize the activity of various promoters in a manner consistent with GFP.

### 3.4 Development of the PLAP‐CoMBI method

**FIGURE 4:**
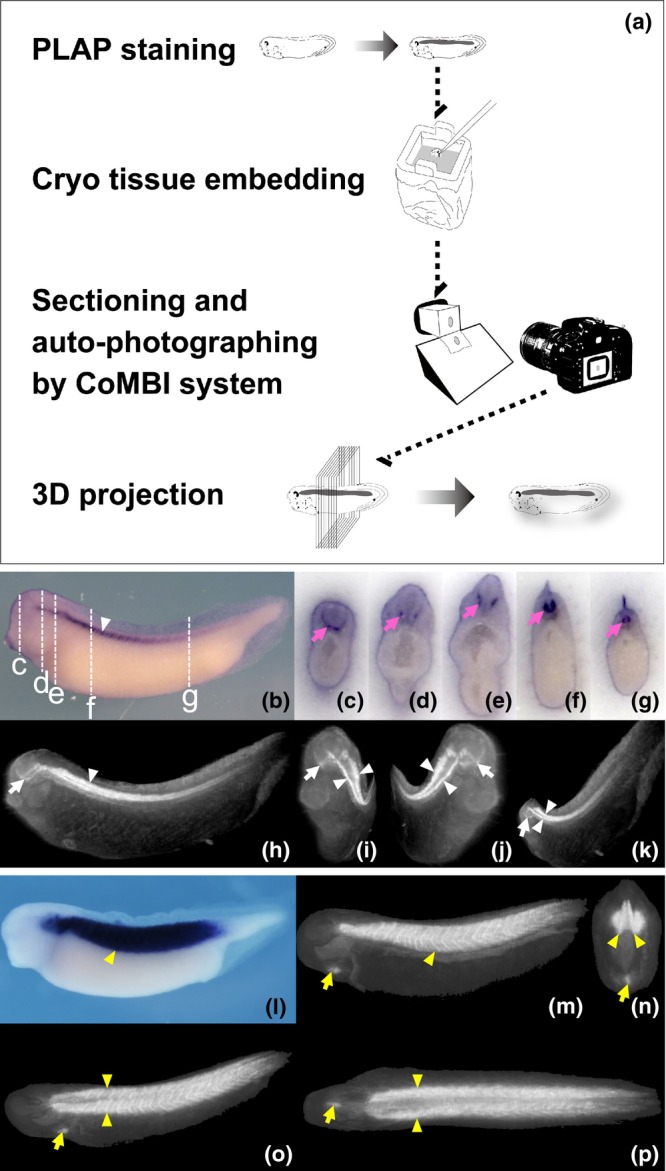
3D imaging analysis by PLAP‐CoMBI. (a) Schematic representation of the experimental procedure. (b) Lateral view of a representative embryo injected with *Tg(Xtr.tubb2b:ALPP)* and analyzed by the CoMBI method. White dotted lines c, d, e, f, and g indicate approximate sectioning planes of block‐face images that are shown in (c–g), respectively. Pink arrows indicate alkaline phosphatase (AP) staining ventral to the forebrain (c) and as two ventrolateral patches each in the hindbrain (d, e) and in the spinal cord (f, g). (h–k) Lateral, left frontal, right frontal, and left posterior views of the 3D image reconstructed from block‐face images of the embryo shown in (b). White triangles and white arrows in (b) and (h–k) indicate AP staining in the spinal cord and in the bridging structure located ventral to the forebrain, respectively. (l) Lateral view of a representative embryo injected with *Tg(Xla.actc1:ALPP)* and analyzed by the CoMBI method. (m–p) Lateral, transverse, left dorsal, and true dorsal views of the 3D image reconstructed from block‐face images of the embryo shown in (l). Yellow triangles and arrows indicate AP staining in the somites and heart primordia, respectively.

The above experiments with the various *cis*‐regulatory elements, including the *six3* enhancer and promoter, have shown that PLAP works as an effective transgenic reporter under the optimized heat treatment condition. Moreover, embryos injected with *Tg(Xtr.tubb2b:ALPP)* exhibited clear AP staining in the spinal cord of tailbud embryos, suggesting that targeted expression of PLAP is very effective in staining deep tissues, owing to the efficient penetration of the AP substrate (stages 33/34, Figure 4b). Thus, we attempted to reconstruct a 3D image of the PLAP‐expressing tissues by analyzing these stained embryos with the CoMBI method. The embryo was embedded in freezing compound and subjected to cryosectioning combined with autophotographing of the serial block‐face images (Figure 4a). Part of the resulting transverse images showed AP staining in a region ventral to the forebrain, as two ventrolateral patches each in the hindbrain and in the spinal cord (Figure 4c–g). Note that the staining ventral to the forebrain was not visible from the surface (Figure 4c, compared with Figure 4b). Then, all the serial images were processed and stacked to reconstruct a 3D image of the PLAP‐expressing tissues using ImageJ v1.47 and Zen 2.6 software, where the AP signals were pseudocolored in black and white (Figure 4h, Movie S1). When this image was rotated horizontally, the PLAP‐expressing tissue was found to form two stripe‐like structures from the hindbrain to the spinal cord, fusing ventral to the forebrain (Figure 4i–k).

In addition to *Tg(Xtr.tubb2b:ALPP)*, we examined the efficacy of the PLAP‐CoMBI method with a reporter construct carrying an *X. laevis actc1* promoter, *Tg(Xla.actc1:ALPP)*. Embryos injected with this construct showed AP staining in the developing somite (Figure 4l), which appears to recapitulate the GFP expression pattern driven by the same promoter element (Latinkić et al., 2002). A lateral view of the 3D image generated by the PLAP‐CoMBI method visualized not only the somite but also the developing heart, whose AP staining was not visible from the surface (Figure 4m, compared with Figure 4l, Movie S2). When this image was rotated horizontally, a pair of left and right somites and the heart primordium were observed in the transverse view (Figure 4n). When the image was rotated vertically, a pair of left and right somites lined through the trunk and the heart primordium were observed in the dorsal view (Figure 4o,p). These results demonstrated the power of PLAP‐CoMBI for the 3D analysis of tissue structures, where deep tissues were visualized in opaque *Xenopus* embryos, and the resulting images were freely rotated horizontally or vertically to investigate their details.

## 4 DISCUSSION

In this study, we optimized the heat treatment condition for detecting exogenous PLAP staining in the absence of endogenous AP‐dependent background staining. Under this optimized condition, we showed that PLAP could visualize the *cis*‐regulatory activities of *six3*, *eef1a1*, *tubb2b*, and *actc1*, in a manner consistent with GFP. In contrast to PLAP, *lacZ* showed highly mosaic expression when linked to the *eef1a1* promoter (Figure 3c). The *lacZ* gene also showed mosaic expression when linked to the *Xenopus foxe3* promoter (data not shown), which can drive lens‐specific expression of GFP (Ogino et al., 2008). Such aberrant *lacZ* expression is likely because of the transcriptional silencing effect on prokaryotic genes introduced into the host chromosomes by transgenesis (MacGregor et al., 1987), since misexpression of *lacZ* by RNA injection is a standard method for cellular lineage labeling in *Xenopus* embryos (Sive et al., 2000). The expression of *lagoZ*, a CpG‐null derivative of *lacZ*, was not mosaic in our transgenic experiments. However, *lagoZ* expression was very weak and restricted to ventral tissues, which were different from the dorsal expression of *lacZ* driven by the same *cis*‐regulatory element (Figure 3c,d). Further analysis is needed to clarify the reason for this.

Most of the previous transgenic studies in *Xenopus* used genes encoding fluorescent proteins as reporters and detected their expression by epifluorescence or chromogenic in situ hybridization analysis with probes specific to the fluorescent protein genes (Kroll & Amaya, 1996; Latinkić et al., 2002; Lerchner et al., 2000; Love et al., 2011; Ogino et al., 2008). Fluorescence analysis is very convenient, but the signal is sensitive to fixation processing and is not permanently preserved. In situ hybridization signals can be preserved semi‐permanently, but the experimental procedure is labor‐intensive and time consuming. Compared with these methods, PLAP staining is much more convenient than in situ hybridization analysis, and resulting chromogenic signals can be preserved semi‐permanently. In addition, PLAP staining appears to be more sensitive than GFP fluorescence as a reporter, owing to the better contrast of AP staining signals than GFP fluorescence in the opaque embryos (Figure 2b). Thus, the PLAP reporter system is expected to facilitate genome‐wide *cis*‐regulatory studies by accelerating high‐throughput transgenic reporter analysis in *Xenopus* embryos.

We combined the PLAP reporter system with the CoMBI method to visualize the 3D structures of deep tissues including the brain, spinal cord, heart primordium, and somite. PLAP‐CoMBI resolves previous problems in 3D imaging of *Xenopus*; efficient probe penetration into deep tissues is not easy for in situ hybridization, and the tissue opacity tends to hinder deep signals in fluorescence analysis. Thus, the use of PLAP‐CoMBI is expected to make *Xenopus* become one of the model vertebrate systems suitable for 3D imaging analysis. Furthermore, this system is a useful tool for identifying novel embryonic tissue structures, as demonstrated by the imaging of the two stripe‐like neural tissues forming a fine bridging structure ventral to the forebrain (Figure 4h–k). This bridging structure may be part of the trigeminal nerves marked by the expression driven by an *X. laevis tubb2b* promoter (Huang et al., 2007). However, further analysis is required to identify this tissue precisely, as the expression driven by the *X. tropicalis tubb2b* promoter in this study may be different from that driven by the *X. laevis tubb2b* promoter in Huang et al. (2007).

## AUTHOR CONTRIBUTIONS

Hajime Ogino designed the research. Kiyo Sakagami, Kaori Saikawa, Yusuke Sakaguchi, Nusrat Hossain, Chiho Kato, Kazuhito Kinemori, Nanoka Suzuki, and Akane Kawaguchi performed experiments. Takeshi Igawa and Yuki Tajika developed the CoMBI system. Takeshi Igawa, Makoto Suzuki, Haruki Ochi, and Hajime Ogino analyzed the data. Hajime Ogino, Takeshi Igawa, and Kiyo Sakagami wrote the manuscript. All authors critically reviewed and revised the first draft and approved the final version for submission.

## Supporting information


**Movie S1.** 3D image of the tissues expressing placental alkaline phosphatase (PLAP) under the control of a *tubb2b* promoter. Screenshots of the movies with different angles are shown in Figure 4h–k.

**Movie S2.** 3D image of the tissues expressing placental alkaline phosphatase (PLAP) under the control of an *actc1* promoter. Screenshots of the movies with different angles are shown in Figure 4m–p.
